# First checklist of mayflies (Insecta, Ephemeroptera) from Kosovo

**DOI:** 10.3897/zookeys.874.38098

**Published:** 2019-09-05

**Authors:** Bardh L. Xërxa, Michel Sartori, Agim Gashi, Jean-Luc Gattolliat

**Affiliations:** 1 Department of Biology, University of Prishtina, 10000 Prishtina, Kosovo University of Prishtina Prishtina Kosovo; 2 Musée Cantonal de Zoologie, Palais de Rumine 6, 1014 Lausanne, Switzerland Musée Cantonal de Zoologie Lausanne Switzerland; 3 Department of Ecology and Evolution, Biophore, University of Lausanne, 1015 Lausanne, Switzerland University of Lausanne Lausanne Switzerland

**Keywords:** Aquatic insects, Balkan Peninsula, freshwater fauna, Kosovo, taxonomy

## Abstract

This research provides the first systematic contribution to the mayfly (Ephemeroptera) Fauna of Kosovo. This investigation was conducted from March to November in 2017 and 2018; 32 sites were sampled covering the different freshwater ecosystems of the country. The first checklist of mayflies of Kosovo is provided. During this survey, we found 48 species belonging to 20 genera and nine families. The highest number of species belongs to the following two families, Heptageniidae (24) and Baetidae (9). This investigation is a contribution to the knowledge about taxonomy, biogeography, and ecology of mayflies of the Balkan Peninsula by giving new data on species composition and distribution range in Kosovo.

## Introduction

Mayflies (Ephemeroptera) are an ancient insect lineage dating back over 300 million years and are believed to be the most primitive group of extant winged insects (Grimaldi and Engel 2005, Bauernfeind and Soldán 2012). Mayflies are merolimnic insects: the larval stage is strictly aquatic, while the imaginal stage is extremely brief and on the wing. Mayflies are able to colonise every kind of freshwater habitat but are mainly diversified in lotic habitats (streams and rivers). They are distributed worldwide with the highest diversity in tropical areas; the order encompasses approximately 3500 species, 450 genera and 42 families (Barber-James et al. 2008, Sartori and Brittain 2015). According to the literature (Bauernfeind and Soldán 2012), 369 species are recorded for Europe and North Africa. Mayflies are considered as keystone species and their presence is believed to be an important environmental indicator of oligotrophic to mesotrophic (i.e., low to moderately productive) conditions in running waters (Barbour et al. 1999, Bauernfeind and Moog 2000). High sensitivity of mayfly taxa to oxygen depletion, acidification, and various contaminants including metals, ammonia, and other chemicals was demonstrated in both observational and experimental studies (Hubbard and Peters 1978, Resh and Jackson 1993, Moog et al. 1997). Various Biological Indices including mayflies to assess water quality have been developed over the years (Lenat 1988, Metcalfe 1989, Kerans and Karr 1994). Subsequently, most of the biological water quality assessment methods for streams include Ephemeroptera, for example, the EPT (Ephemeroptera + Plecoptera + Trichoptera) taxa richness (Lenat and Penrose 1996).

Faunistics and taxonomy of mayflies in the Balkans is still in progress and the level of knowledge varies between different countries. Neighbouring mayfly fauna is relatively well known, mostly thanks to studies in Croatia (Vilenica et al. 2015), N. Macedonia (Ikonomov 1961a, 1961b, 1962, 1963, 1964), Serbia (Petrovic et al. 2014, Slovenia (Zabric and Sartori 1997), Bosnia and Hercegovina (Bauernfeind and Soldán 2012), Bulgaria (Vidinova 2003, Vidinova and Janeva 2000, Vidinova and Russev 1997, Vidinova et al. 2006), and Hungary (Kovács and Bauernfeind 2003). By contrast, fewer than ten species are currently known from Albania (Kovács and Murányi 2013) reflecting insufficient research effort in this country.

**Figure 1.**
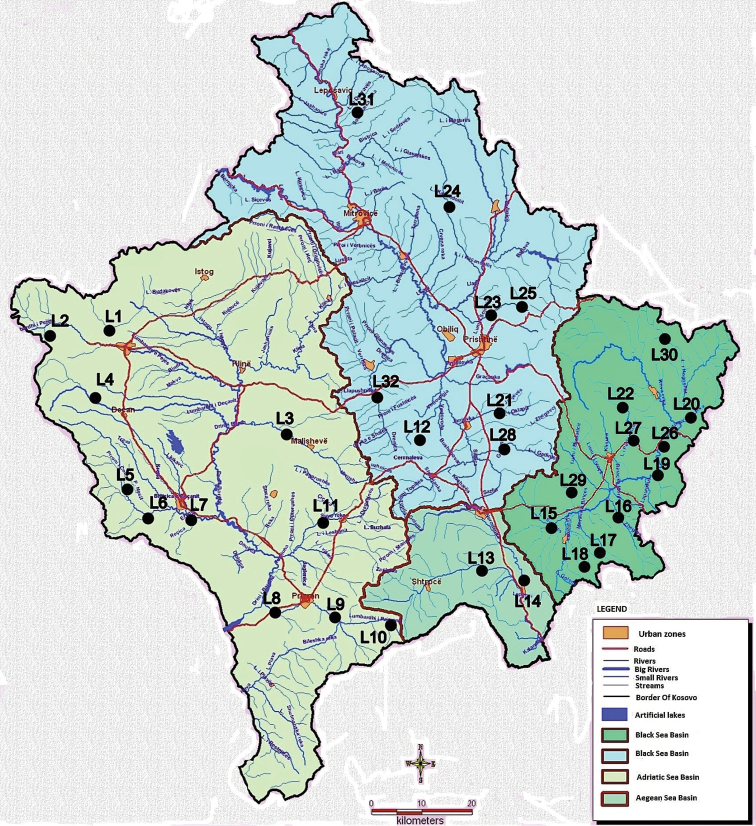
Map of Kosovo indicating the collection sites.

Kosovo is a small landlocked country in the centre of Balkan Peninsula and is divided into two ecoregions: Dinaric Western Balkan (ER5) and Hellenic Western Dinaric (ER6) (Illies 1978). In hydrographical terms, Kosovo is divided into four river basins: Drini i Bardhë, Ibri, Morava e Binçës, and Lepenci which flow into three sea basins: Black Sea, Adriatic Sea, and Aegean Sea (Fig. 1). Kosovo has a total area of 10,908 km² with an altitude range from 265 m to 2656 m. The mountains of Kosovo belong to the Dinarides range with two major mountain massifs, Sharr and Bjeshkët e Nemuna.

Published data on mayflies from Kosovo are scarce. However, as a part of former Yugoslavia, there are some records published by different authors: Puthz 1974 (*Baetis alpinus*, *Baetis rhodani*, *Epeorus assimilis* (wrongly identified as *Epeorus sylvicola* (Pictet, 1865)), *Ecdyonurus insignis*, *Rhithrogena germanica*, and *Ephemerella krieghoffi* (Ulmer, 1920) (now considered as a junior synonym of *Ephemerella mucronata*); Studemann et al. 1989 (*Ephemerella ikonomovi* species now assigned to *Quatica*); Hefti and Tomka 1988 (*Ecdyonurus subalpinus*). Other papers only explored mayflies as part of the general macroinvertebrate assemblage, which is essential for the implementation of bioassessment tools for the local stream systems (Shukriu 1979, Gashi 1993, 2005, Zhushi-Etemi 2005, Kuqi 2006, Ibrahimi 2007). Consequently, little data was available about the mayfly fauna of Kosovo and no major collections have been housed so far. The distribution range of the different species throughout the territory remains unknown. Therefore, in this paper, we include the first checklist of species and the distribution of mayfly species in Kosovo.

## Materials and methods

### Sampling and laboratory methods

Most of the studied material for this research was collected during the two-year sampling period from March to November 2017/2018. Mayflies were sampled every month at 21 sites while at the remainder of sites, sampling was usually performed only once during this time. Specimens were collected in freshwater habitats (mainly lotic and some lentic) in over 32 sites throughout Kosovo’s territory (Fig. 1).

The detailed list of the 32 sampling site names with number codes (site ID), altitude, latitude and longitude are presented in Table 1. Mayfly larvae were collected using a hand D-net or picked manually from rocks and pebbles, while imagos were caught with a hand net and light traps, then preserved in 96 % ethanol. Collected specimens were studied under a Leica M205 and Olympus stereomicroscope.

**Table 1.** Sampling site characteristics.

Code	Sampling site	Altitude	Latitude / Longitude	Ecoregion	Sea basin	Habitat
**L1**	Radavcë	1170 m	42°44.14'N, 20°18.51'E	ER5 (Dinaric)	Adriatic	Lotic
**L2**	Çakorr	1242 m	42°41.31'N, 20°04.38'E	ER5 (Dinaric)	Adriatic	Lotic
**L3**	Mirusha	370 m	42°31.25'N, 20°34.50'E	ER5 (Dinaric)	Adriatic	Lotic
**L4**	L. Deçanit-Manastiri	903 m	42°54.71'N, 20°26.66'E	ER5 (Dinaric)	Adriatic	Lotic
**L5**	L. Erenik-Botusha	874 m	42°30.00'N, 20°14.47'E	ER5 (Dinaric)	Adriatic	Lotic
**L6**	L. Erenik-Devë	567 m	42°28.42'N, 20°16.53'E	ER5 (Dinaric)	Adriatic	Lotic
**L7**	L. Erenik-Travë	345 m	42°22.32'N, 20°24.15'E	ER5 (Dinaric)	Adriatic	Lotic
**L8**	L. Prizren-Vlashnje	364 m	42°10.02'N, 20°31.05'E	ER6 (Hellenic)	Adriatic	Lotic
**L9**	Prizren-Reçan	532 m	42°17.03'N, 21°21.74'E	ER6 (Hellenic)	Adriatic	Lotic
**L10**	Prevall	1664 m	42°16.10'N, 20°95.33'E	ER6 (Hellenic)	Adriatic	Lotic
**L11**	Piran (L.Toplluha)	394 m	42°28.81'N, 20°67.17'E	ER6 (Hellenic)	Adriatic	Lotic
**L12**	Blinaja	721 m	42°51.85'N, 20°97.88'E	ER6 (Hellenic)	Black	Lentic
**L13**	Shtërpcë (Brod)	692 m	42°16.26'N, 21°07.73'E	ER6 (Hellenic)	Aegean	Lotic
**L14**	Nerodime E. Jezercë	810 m	42°21.22'N, 21°01.14'E	ER6 (Hellenic)	Aegean	Lotic
**L15**	Viti	520 m	42°30.62'N, 21°36.20'E	ER6 (Hellenic)	Black	Lotic
**L16**	Mbi Zhegër	660 m	42°29.51'N, 21°54.58'E	ER6 (Hellenic)	Black	Lotic
**L17**	Stanqiq	800 m	42°25.50'N, 21°55.02'E	ER6 (Hellenic)	Black	Lotic
**L18**	Lugu i Kopilaqës	1175 m	42°24.60'N, 21°43.11'E	ER6 (Hellenic)	Black	Lotic
**L19**	Sanakov	625 m	42°25.90'N, 21°34.33'E	ER6 (Hellenic)	Black	Lotic
**L20**	Letnicë	662 m	42°28.72'N, 21°45.73'E	ER6 (Hellenic)	Black	Lotic
**L21**	Slivovë	646 m	42°36.70'N, 21°18.19'E	ER6 (Hellenic)	Black	Lotic
**L22**	Binçë (Debelldeh +Buzovik)	566 m	42°29.48'N, 21°37.17'E	ER6 (Hellenic)	Black	Lotic
**L23**	Mramor	635 m	42°37.84'N, 21°16.47'E	ER5 (Dinaric)	Black	Lotic
**L24**	Keçekoll	754 m	42°43.45'N, 21°18.50'E	ER5 (Dinaric)	Black	Lotic
**L25**	Makovcë	626 m	42°41.92'N, 21°14.23'E	ER5 (Dinaric)	Black	Lotic
**L26**	Dermjak	606 m	42°17.22'N, 21°31.57'E	ER6 (Hellenic)	Black	Lotic
**L27**	Stanishor	622 m	42°36.70'N, 21°18.19'E	ER6 (Hellenic)	Black	Lotic
**L28**	Shushtë-Kabash	525 m	42°28.11'N, 21°35.91'E	ER6 (Hellenic)	Black	Lotic
**L29**	Mjak	735 m	42°25.36'N, 21°34.76'E	ER6 (Hellenic)	Black	Lotic
**L30**	Korbiliq	730 m	42°22.98'N, 21°33.58'E	ER6 (Hellenic)	Black	Lotic
**L31**	Ibri-Leposaviç	544 m	42°59.66'N, 20°48.97'E	ER5 (Dinaric)	Black	Lotic
**L32**	Ligatina e Hencit	545 m	42°51.85'N, 20°97.88'E	ER5 (Dinaric)	Black	Lentic

Mayflies were found at all 32 sampled localities (Fig. 1). The majority of specimens were collected at the larval stage, but a small number of adults were caught in the field also. The identification of the mayflies to species level has been performed by using the books by Bauernfeind and Humpesch (2001), Eiseler (2005), Bauernfeind and Soldán (2012), Elliott et al. (1988), and Soldán and Landa (1999); when necessary, morphological characters were checked in the original descriptions (Braasch 1984, Jacob 1974, Jacob and Braasch 1984, 1986, Puthz 1971, Ikonomov 1961a, and Demoulin 1958). Nomenclature and family assignment follow Bauernfeind and Soldán (2012), except for the family Ephemerellidae for which we follow Jacobus and McCafferty (2008) and Baetidae for which we consider *Alainites* and *Procloeon* as valid genera. As part of the species is still incompletely described or one of the two stages remains unknown, the association of larval and adult stages is sometimes challenging in mayflies. Without rearing in the field, the use molecular data such as mitochondrial gene (the animal “barcode”) will provide an alternative for association of ontogenetic stages. For these reasons, identification of some species remains difficult; they are therefore referred to as cf. or as sp. All material examined are housed in the Museum of Zoology, Lausanne, Switzerland, and Laboratory of Faculty of Natural Sciences, Pristina, Kosovo. Authorships of the species are indicated in Table 3; they are not mentioned in the main text, except for species not occurring in Kosovo.

## Results

The current research produced the first comprehensive contribution of mayfly taxa inhabiting Kosovo’s freshwater habitats. In 32 sampling stations, a total of 7564 individuals of mayfly larvae and adults was collected in rivers, streams and some lentic habitats. Sampling sites included a wide range of elevation with lowest L7 at 345 m and highest L10 at 1664 m (Table 1).

Based on the analysed data, in total, 48 species distributed into 20 genera and nine families were recorded (Table 2). The most diversified family was Heptageniidae (four genera and 24 species), followed by Baetidae (four genera and nine species). The following families had only one species: Ameletidae, Oligoneuriidae, and Potamanthidae. The most diverse genera were *Ecdyonurus* with 13 species, *Rhithrogena* with seven species, and *Baetis* with five species. Seven species were recorded at only one site: *Potamanthus luteus*, Electrogena cf. mazedonica, Procloeon cf. pulchrum, Ecdyonurus cf. siveci, *Ephemera vulgata*, *Caenis horaria* and Caenis cf. strugaensis.

**Table 2.** Kosovo Ephemeroptera composition.

Family	Number of genera	% Genus	Number of species	% Species
Ameletidae	1	5.00	1	2.08
Baetidae	4	20.00	9	18.75
Oligoneuriidae	1	5.00	1	2.08
Heptageniidae	4	20.00	24	50.00
Leptophlebiidae	3	15.00	3	6.25
Potamanthidae	1	5.00	1	2.08
Ephemerellidae	4	20.00	4	8.33
Ephemeridae	1	5.00	2	4.16
Caenidae	1	5.00	3	6.25
	**20**	**100**	**48**	**100**

The most frequently encountered species was *Baetis rhodani* which was recorded from 21 of the 32 sites. *Baetis alpinus* and Ephemera cf. parnassiana were found at 14 and seven different sites, respectively (Table 3.). Three species were recorded as adults only at one site: *Ecdyonurus graecus* (site L20), Ecdyonurus cf. puma (site L16) and *Paraleptophlebia submarginata* (site L13). The remaining species occurred at between two to six sites. Taxa richness per locality varied from two to 17 species.

The highest taxa richness was found along sampling site L21-Slivovë (17 species) and L12-Blinajë (16 species) and the lowest species richness (two species) was observed at site L8. Of the total mayfly species (48) for the three Sea Basins, 42 species were discovered for the Black Sea Basin, 29 species for the Adriatic Sea Basin, and only two species in the Aegean Sea Basin.

## Discussion

Due to the absence of consistent data and research on mayfly fauna as well as of their habitat preferences in Kosovo, this study provides the first global contribution to the mayflies of Kosovo with 48 recorded mayfly taxa. However, out of 48 species, three were previously recorded and not found in the present study: by Puthz (1974) two species (*Ecdyonurus insignis*, *Rhithrogena germanica*) and by Hefti and Tomka (1988) one species (*Ecdyonurus subalpinus*) (Table 3). Most of the species collected during this investigation belong to the Western-Palearctic group with 23 followed by the Balkan group with seven species, the Holarctic group with six species, the South Europe group with six species, the Palearctic group with four species, and the Holomediterranean group with two species.

**Table 3.** Kosovo mayfly fauna: first checklist of species with distribution. Key: NR: new records for Kosovo; ▲: data from literature only (Puthz 1974, Hefti and Tomka 1988).

Ephemeroptera taxa		Adriatic Sea basin	Black Sea basin	Aegean Sea basin
**Family: Ameletidae** McCafferty, 1991				
**Genus: *Metreletus*** Demoulin, 1951				
1. *Metreletus balcanicus* (Ulmer, 1920)	NR		L12, L24	
**Family: Baetidae Leach, 1815**				
**Genus: *Baetis*** Leach, 1815				
2. *Baetis rhodani* (Pictet, 1843)		L3–L11	L12, L15, L16, L17, L20, L21, L23, L24, L25, L28, L29, L30	
3. *Baetis alpinus* (Pictet, 1843)		L1, L2, L3, L4, L5, L6, L7, L9, L10	L28, L29, L30	L13, L14
4. *Baetis buceratus* Eaton, 1870	NR	L3	L16, L21, L23	
5. *Baetis melanonyx* (Pictet, 1843)	NR	L4, L5, L6, L9		L13, L14
6. *Baetis pentaphlebodes* Ujhelyi, 1966	NR		L12, L21, L32	
**Genus: *Alainites*** Waltz & McCafferty, 1984				
7. *Alainites muticus* (Linnaeus, 1758)	NR	L1, L5, L6	L32	
**Genus: *Cloeon*** Leach, 1815				
8. *Cloeon dipterum* (Linnaeus, 1761)	NR	L11	L12, L32	
9. Cloeon cf. dipterum (Linnaeus, 1761)	NR		L12, L32	
**Genus: *Procloeon*** Bengtsson, 1915				
10. Procloeon cf. pulchrum (Eaton, 1885)	NR		L12	
**Family: Oligoneuriidae** Ulmer, 1914				
**Genus: *Oligoneuriella*** Ulmer, 1924				
11. *Oligoneurella rhenana* (Imhoff, 1852)	NR	L6, L7	L21	
**Family: Heptageniidae Needham, 1901**				
**Genus: *Epeorus*** Eaton, 1881				
12. *Epeorus assimilis* Eaton, 1885		L6, L9	L25, L28	
13. *Epeorus yougoslavicus* (Šamal, 1935)	NR	L2, L9		
**Genus: *Ecdyonurus*** Eaton, 1871				
14. *Ecdyonurus graecus* Braasch, 1984	NR	L1	L20, L30	
15. Ecdyonurus cf. epeorides Demoulin, 1955	NR	L3	L21, L22, L26, L27	
16. Ecdyonurus cf. puma Jacob & Braasch, 1986	NR		L16, L17	
17. *Ecdyonurus macani* Thomas & Sowa, 1970	NR	L1	L12, L21	
18. *Ecdyonurus starmachi* Sowa, 1971	NR	L3	L12, L19, L21, L22	
19. *Ecdyonurus vitoshensis* Jacob & Braasch, 1984	NR		L18, L24, L31	
20. *Ecdyonurus venosus* (Fabricius, 1775)	NR	L1, L3	L15, L21, L20, L24	
21. *Ecdyonurus submontanus* Landa, 1969	NR	L3, L5	L22	
22. Ecdyonurus cf. krueperi (Stein, 1863)	NR		L21, L22	
23. Ecdyonurus cf. siveci Hefti, Tomka & Zurwerra, 1986	NR	L2		
24. *Ecdyonurus* sp.			L12, L21	
25. *Ecdyonurus insignis* (Eaton, 1870)	▲	–	–	–
26. *Ecdyonurus subalpinus* (Klapálek, 1907)	▲	–	–	–
**Genus: *Rhithrogena*** Eaton, 1881				
27. *Rhithrogena braaschi* Jacob, 1974	NR	L2, L9	L21	
28. Rhithrogena gr. sowai Puthz, 1972	NR	L9	L21	
29. Rhithrogena cf. bulgarica Braasch, Soldán & Sowa, 1985	NR	L9	L16, L25, L28, L29, L30	
30. Rhithrogena gr. hercynia Landa, 1969	NR	L9	L25	
31. Rhithrogena gr. semicolorata (Curtis, 1834)	NR	L9, L10	L25	
32. Rhithrogena gr. diaphana Navàs, 1917	NR	L9	L15, L21, L24	
33. *Rhithrogena germanica* Eaton, 1885	▲	–	–	–
**Genus: *Electrogena*** Zurwerra & Tomka, 1985				
34. Electrogena cf. mazedonica (Ikonomov, 1954)	NR		L12	
35. *Electrogena* sp.		L11	L12	
**Family: Leptophlebiidae (Banks, 1900)**				
**Genus: *Habrophlebia*** Eaton, 1881				
36. *Habrophlebia eldae* Jacob & Sartori, 1984	NR		L12, L24	
**Genus: *Habroleptoides*** Schoenemund, 1929				
37. *Habroleptoides confusa* Sartori & Jacob, 1986	NR	L5, L6	L21, L23, L25	
**Genus: *Paraleptophlebia*** Lestage, 1917				
38. *Paraleptophlebia submarginata* (Stephens, 1836)	NR		L13, L15, L20, L21, L28	
**Family:Potamanthidae Albarda, 1888**				
**Genus: *Potamanthus*** Pictet, 1843				
39. *Potamanthus luteus* (Linnaeus, 1767)	NR		L20	
**Family: Ephemerellidae Klapálek, 1909**				
**Genus: *Torleya*** Lestage, 1917				
40. *Torleya mayor* (Klapalek, 1905)	NR	L11	L21, L25, L28	
**Genus: *Serratella*** Edmunds, 1959				
41. *Serratella ignita* (Poda, 1761)	NR	L3, L5, L6, L8, L11	L21	
**Genus: *Quatica*** Jacobus & McCafferty, 2008				
42. *Quatica ikonomovi* (Puthz, 1971)		L8, L9	L12, L15	
***Genus: Ephemerella*** Walsh, 1863				
43. *Ephemerella mucronata* (Bengtsson, 1909)	NR	L11	L32	
**Family: Ephemeridae Latreille, 1810**				
**Genus: *Ephemera*** Linnaeus, 1758				
44. Ephemera cf. parnassiana Demoulin, 1958	NR		L12, L17, L20, L21, L23, L24, L29	
45. *Ephemera vulgata* Linnaeus, 1758	NR		L12	
**Family**: **Caenidae** Newman, 1853				
**Genus: *Caenis*** Stephens, 1836				
46. *Caenis macrura* (Stephens, 1835)	NR	L11	L12, L32	
47. *Caenis horaria* (Linnaeus, 1758)	NR		L12	
48. Caenis cf. strugaensis Ikonomov, 1961	NR		L12	

In comparison with the neighbouring countries and with consideration of their surface areas, the recorded Ephemeroptera diversity in Kosovo could be characterised as intermediate. The highest number of species was listed for Bulgaria with 102 taxa (Vidinova 2003), Serbia with 85 taxa (Petrovic et al. 2014, Croatia with 79 taxa (Vilenica et al. 2015) followed by Slovenia with 75 taxa (Zabric and Sartori 1997), N. Macedonia with 63 taxa (Smith and Smith 2003), and Bosnia and Hercegovina with 51 taxa (Bauernfeind and Soldán 2012). In this research, collecting was carried out mainly in running waters; therefore, lentic species are less diversified. Nonetheless, it was discovered that most of the Kosovo mayfly species are associated with rivers and streams. Some species of the genera *Ecdyonurus* and *Rhithrogena* are still considered as cf. and gr. (Table 3) because of the uncertainty of identifications as well as species unknown or poorly known at only one stage. *Metreletus balcanicus*, a rare European mayfly species, was recorded in two sites (L12 and L24) with low numbers of individuals; consequently, it could be considered a rare species in Kosovo too. According to the Fauna Europaea database, the species is present in Bulgaria, Czech Republic, Germany, Luxemburg, French Mainland, Hungary, Poland, and the European part of Turkey (de Jong et al. 2014). Balkan endemics (Electrogena cf. mazedonica, *Rhithrogena braaschi*, and Ephemera cf. parnassiana) were also recorded. Regarding the species Electrogena cf. mazedonica, it is a rare Balkan endemic with records in Macedonia (Ikonomov 1964) and provisional records from the northern border of Greece (Bauernfeind 2003). Our findings show it is a rare species also in Kosovo with records in only one sampling station (L12). *Rhithrogena braaschi* has probably a Pontic origin, recorded so far from the Balkans: Bulgaria (e.g., Vidinova et al. 2006) and Greece (Bauernfeind 2003). In our collections, it occurred in three localities: L2, L9, and L21. Ephemera cf. parnassianais a very rare Balkan endemic species present only in Greece (Bauernfeind 2003) and Croatia (Vilenica et al. 2015); nonetheless, in Kosovo, it had a wider distribution with a large number of individuals.

During our research, the highest number of species (17) were recorded from sample site L21-Slivovë and 16 species from the L12-Blinajë. On the other hand, high elevation sites (L2, L10, and L18) had the lowest number of mayfly species (two) as well as one sample site L8 at a low elevation with only two species. Sample sites L12 and L21 were high in species diversity because they were covered with macrophytic vegetation and different substrates in a clean habitat with altitudes of approximately 700 m. On the other hand, L8 was low on species diversity because it is affected by pollution from outside the large town and is well subjected to long-term anthropogenic stress from discharged urban sewage. The majority of Kosovo mayflies belong to the south European, central European, and Mediterranean faunas. For each species, their geographical distribution is presented as well as the sample site at which it was collected (Tables 1, 3).

The new records include some morphologically interesting taxa and difficult complex of species (Cloeon gr. dipterum, Rhithrogena gr. sowai, and Ecdyonurus gr. venosus). As two of the most similar mayfly assemblages of the neighbouring countries (N. Macedonia, Serbia) have several taxa that could also inhabit Kosovo habitats (e.g., *Baetis vardarensis* Ikonomov, 1962, *Baetis liebenauae* Keffermüller, 1974, *Cloeon simile* Eaton, 1870), but were not yet recorded, due to the lack of regular sampling in all seasons, future research should include seasonal sampling of a higher number of sites and habitat types. Further study is required at new sampling sites to determine the distribution of seven species recorded only at a single sampling site (*Potamanthus luteus*, Electrogena cf. mazedonica, Procloeon cf. pulchrum, Ecdyonurus cf. siveci, *Ephemera vulgata*, *Caenis horaria*, and Caenis cf. strugaensis).

Mayflies are generally diverse in lotic ecosystems as the majority of species prefer well-oxygenated habitats (Merritt et al. 2008). Consequently, the highest species diversities in this study were recorded along rivers and streams. The richest genera were *Ecdyonurus* (13 species), *Rhithrogena* (seven species), and *Baetis* (five species), which are known to be very prevalent in running waters of the northern hemisphere (Bauernfeind and Soldán 2012). *Baetis rhodani* was the most commonly encountered taxon in Kosovo and occurred in 66 % of the sampled sites. This species was found at a variety of lotic habitats including rivers and streams. The elevation range of this species in Kosovo extended from 400 to 1000 m. The wide occurrence of this species among our sampled sites is most likely due to its very broad ecological range (Bauernfeind and Soldán 2012). However, several habitats have been poorly investigated, such as those at high altitudes above 1800 m.

The present study is a significant contribution to the understanding of the mayfly fauna in Kosovo and the Balkan Peninsula, with the country’s first checklist along with some rare species records. Therefore, the current study adds to a stronger knowledge of Kosovo’s mayfly fauna and may promote the development of regional biological water quality indicators.

Some interesting taxa with restricted European and local distributions were recorded (e.g., Rhithrogena cf. bulgarica, *Metreletus balcanicus*, and *Epeorus yugoslavicus*). Considering these species were collected in a limited number of sites in this study, they could be considered as rare. Future studies on the conservation status and ecological features of these species are necessary.

## Conclusion

As there was essentially no systematic research on mayfly fauna (species diversity and distribution) in Kosovo, this research is the first contribution toward mayfly inventory of this part of the Balkan Peninsula based on larvae and adult specimen collections. Kosovo’s mayfly fauna comprises 9 families, 20 genera, and 48 species. Out of 48 mayfly taxa, 45 species are new records from Kosovo. The present research gives the record of Kosovo mayflies which belong to the West Palearctic, Central South European, Balkan, and Mediterranean species. Of 48 taxa, approximately half of the species were present in both Ecoregions (ER5 and ER6). Nonetheless, several habitats have been poorly investigated, such as high altitude habitats (above 1800 m). Further, emphasis on lentic habitats will be made as taxa such as *Caenis* spp. and *Cloeon* spp. are still under-sampled. Therefore, this research constitutes a first contribution to mayfly fauna of Kosovo and is far from complete.

The updating of this first mayfly checklist is highly expected with new investigations. Furthermore, the recorded diversity of Ephemeroptera in Kosovo could be defined as intermediate compared to neighbouring countries, taking into account their surface areas. The highest diversity was observed in submontane regions, while the lowest was detected in rivers and the majority of species collected in this research belong to grazers–scrapers and gatherers–collectors. The future challenges will be to identify the cryptic species within a difficult complex of species (Cloeon gr. dipterum, Rhithrogena gr. sowai, and Ecdyonurus gr. venosus) with careful taxonomical examination and the use of DNA barcoding.

This first checklist of mayflies and their distribution are intended to serve as a foundation and stimulation for further research since the records of many species and their distribution patterns within Kosovo can surely be amended in the future. Moreover, given the high diversity of freshwater habitats within four river basins and the scarce research on mayflies, finding species new for the country (or even new to science) are highly expected.

Finally, further research could clarify the ecological preferenda of each species and their degree of vulnerability in Kosovo to offer an essential tool for running water management and river quality assessments. New knowledge about the Ephemeroptera diversity and distribution in Kosovo will be highly beneficial for further investigation and biomonitoring of the environmental changes in freshwater habitats including the evaluation of other anthropogenic impacts.
